# Crashing Left vs. Right: Examining Navigation Asymmetries Using the SHRP2 Naturalistic Driving Study Data

**DOI:** 10.3389/fpsyg.2017.02153

**Published:** 2017-12-12

**Authors:** Trista E. Friedrich, Lorin J. Elias, Paulette V. Hunter

**Affiliations:** ^1^Department of Psychology, University of Saskatchewan, Saskatoon, SK, Canada; ^2^Department St. Thomas More College, University of Saskatchewan, Saskatoon, SK, Canada

**Keywords:** aging, pseudoneglect, navigation asymmetry, attention, naturalistic driving

## Abstract

The magnitude of leftward bias demonstrated in pseudoneglect has been found to differ between younger and older adults in laboratory settings. The objective of this study was to examine the association between age and asymmetries in navigation in a naturalistic setting by examining the frequency of the location of impact on participants' vehicles during crashes and near crashes. The location of impact following crashes and near crashes, and participant's age and gender were retrieved from the SHRP2 NDS database, a large scale naturalistic driving study. Over the course of the study, data were collected from 3,546 participants driving in the United States of America (right-side traffic directionality), which included 1,465 crashes and 2,722 near crashes. During crashes and near crashes, irrespective of age, the location impact was most often on the front side of the participant vehicle. In contrast with results from laboratory environments, age was not associated with the location of impact during crashes and near crashes, and overall, crashes were over-represented on the left side of the vehicle compared to the right. Specifically, crashes were 1.41 times as likely to occur on the left compared to the right side of participants' vehicles. Overall, these findings inform future research that attempts to apply laboratory research, regarding asymmetry in navigation, to naturalistic settings.

## Introduction

Estimating the center between two points to avoid a collision is a seemingly simple task that is required to complete many everyday activities. These activities include walking through doorways and crowds of people, parking vehicles, and taxing an airplane. Nevertheless, laboratory researchers who study simple tasks that require estimating the center, such as bisecting lines, document a small but consistent bias to the left side of space among neurologically healthy individuals (Nicholls et al., 1999; Jewell and McCourt, 2000). This bias to the left hemifield within peripersonal space is a robust phenomenon known as pseudoneglect (Bowers and Heilman, 1980), and has been demonstrated in a variety manual bisection and perceptual tasks.

Although pseudoneglect is widely considered to be a systematic bias in attention to the left side of space (i.e., midpoint estimations deviate to the left of the true center; Jewell and McCourt, 2000), research examining pseudoneglect in older adults documents apparent inconsistencies. Some researchers have identified an attenuation of the leftward bias with age, some have identified a rightward bias (i.e., perception that midpoint estimations deviate to the right of true center), and still others have found that older adults have a stronger leftward bias compared to younger adults. For example, a shift from a leftward bias to a rightward bias with age has been demonstrated using the line bisection task (Fukatsu et al., 1990; Fujii et al., 1995; Failla et al., 2003; Barrett and Craver-Lemley, 2008; Goedert et al., 2010; Chen et al., 2011) and landmark task (Schmitz and Peigneux, 2011; Benwell et al., 2014). In contrast, the reverse pattern, a stronger leftward bias with age, has been demonstrated using the line bisection task (De Agostini et al., 1999; Beste et al., 2006; Varnava and Halligan, 2007; Hatin et al., 2012), landmark task (Harvey et al., 1995), tactile rod bisection task (Brooks et al., 2011), and grayscales task (Mattingley et al., 2004; Friedrich et al., 2016).

A number of models have been proposed to account for age related changes in pseudoneglect. These models support the attenuation of the leftward bias with age. The hemispheric asymmetry reduction in older adults (HAROLD) model proposes that aging is associated with a decrease in lateralized activity in frontal regions that results from recruitment or reduced inhibition of the left (non-dominant) hemisphere to compensate for impairment in the right hemisphere (Cabeza, 2002). During visuospatial tasks, activation of the left hemisphere results in lateralization of pertinent features to the right and an absence or reversal of pseudoneglect. Similarly, the right hemi-aging model (RHAM), suggests that the right hemisphere is more sensitive to aging, resulting in a reduction of attentional inhibitory mechanisms (Chieffi et al., 2014), and a more pronounced decline in right hemisphere dominant cognitive functions including spatial processing (Dolcos et al., 2002). Reduced arousal and down-regulation of the attention network in the right hemisphere is suggested to be related to change in dopamine neurotransmission (Midgley and Tees, 1986; Ebersbach et al., 1996; Greene et al., 2010). Dopamine transporter density has been shown to decrease with age (Lavalaye et al., 2000), which may also account for a rightward shift in attentional biases across the lifespan.

Beyond peripersonal space, pseudoneglect has also been associated with tasks that involve extrapersonal space, such as navigating through one's environment. In contrast to the modest leftward bias identified during manual bisection perceptual tasks, a subtle rightward asymmetry during navigation has been found when participants interact with their environment (Turnbull and McGeorge, 1998; Nicholls et al., 2007, 2008, 2010, 2016; Jang et al., 2009; Robertson et al., 2015). The investigation of asymmetry in navigation was initiated by Turnbull and McGeorge (1998) who used a self-report design to inquired about participants' recent collisions with objects, and the side of the body that he or she collided with. Participants tended to report a greater number of collisions on the right side of their body and those who collided on the right demonstrated larger deviations to the left of center on the line bisection task (Turnbull and McGeorge, 1998). Turnbull and McGeorge (1998) suggested that individuals who demonstrate a stronger leftward bias are less likely to attend to the right hemispace and, as a result, have a greater number of rightward collisions. The behavioral effect of lateral attention, the collisions, were presumed to be associated with pseudoneglect and analog to the behavior demonstrated by patients with hemi-spatial neglect (Turnbull and McGeorge, 1998). Similarly, laboratory-based experiments have found predominant right-sided veering and collisions when walking through a narrow doorway (Nicholls et al., 2007), as well as a correlation between bumping and line bisection. Specifically, individuals who bumped the right of the doorway had a larger leftward bias on the line bisection task (Nicholls et al., 2008).

Rightward veering, ranging from 10 to 36 mm, and rightward deviation in navigation has also been demonstrated when navigating an electric wheelchair and scooter through a doorway (Nicholls et al., 2010; Robertson et al., 2015). Similarly, rightward veering and collisions have been found and among participants driving a miniature remote vehicle, particularly when navigating through wider apertures (Nicholls et al., 2016), and while driving a car in a driving simulator (Jang et al., 2009). Although the rightward deviations reported are small, systematic asymmetries in navigation are important to note, as they can lead to inaccurate perceptual judgments and collisions (Nicholls et al., 2016).

Very few studies have extended research on age-related changes in pseudoneglect to the investigation of asymmetry in navigation, nor have many studies extended laboratory-based research on navigational asymmetries to naturalistic settings where participants have greater task demands and navigation is more complicated. Driving is a complex task that requires controlling an approximately 3,000-pound projectile while navigating road, traffic, pedestrians, and technology demands. Attentional lapses and deviations have devastating consequences. In 2013, motor vehicle collisions account for 1,923 deaths and 10,315 serious injuries in Canada (Transport Canada, 2015), and cost approximately 2–3% of the country's Gross Domestic Product (World Health Organization, 2004). Further, developed countries have rapidly-aging populations (Cohen, 2003) resulting in a growing number of older drivers. Hence, it is of interest to examine the association between aging and asymmetries in navigation during motor vehicle collisions.

Among the most common methods used to analyze motor vehicle collisions are self-report, epidemiological data (e.g., crash databases, police reports), and empirical data from driving simulators and driving courses (Klauer et al., 2006). However, the most significant shortcoming of these approaches is that the data can only be said to approximate true driving behavior. In addition, a full picture of the context surrounding a crash incident is typically missing, since data focuses on very specific periods surrounding crash incidents (i.e., pre or post-crash; Klauer et al., 2006). A method that addresses these shortcomings is a large-scale naturalistic driving study, which allows for direct and more complete examination of driver behavior, driving performance, and the relationship between these variables. Further, naturalistic driving studies take place in a naturalistic setting, which enhances the external validity of the study and minimizes the influence of factors associated with the awareness of participation (Carsten et al., 2013). Driving behavior is observed by installing unobtrusive instrumentation devices [e.g., global positioning system (GPS), high frequency cameras, radar] directly linked to vehicle inputs (e.g., steering, breaking, acceleration) from key-on to key-off (Shankar et al., 2008). The instrumentation techniques employed in naturalistic driving studies allow researchers to monitor driving behaviors and kinematic signatures, and detect critical-incident events in a manner that is quantifiable and objective (Manning and Schultheis, 2013). The collection of objective pre-crash information is particularly valuable as it can complement previous research observed in laboratory environments and generate new hypotheses that can be tested under controlled conditions (Carsten et al., 2013).

The recently completed second Strategic Highway Research Program (SHRP 2) is the largest naturalistic driving study (NDS) of its kind. The study included approximately 3,500 participants (16–98 years) from six states across the United States of America (Florida, Indiana, North Carolina, New York, Pennsylvania, and Washington). Participants' personal vehicles were instrumented with a Next Generation data acquisition system (DAS) that included multiple camera views, GPS, speedometer, three-dimension accelerometer and rate sensor, forward radar, illuminance and passive cabin alcohol presence sensors, turn signal state, vehicle network data, and an incident push button. Over the course of 12–24 months, driving data collected from participants encompassed 35 million vehicle miles and consumed two petabytes (PB) of storage space. A detailed description of the study recruitment, participants, and methodology is outlined in Antin et al. (2011).

**Figure 1:**
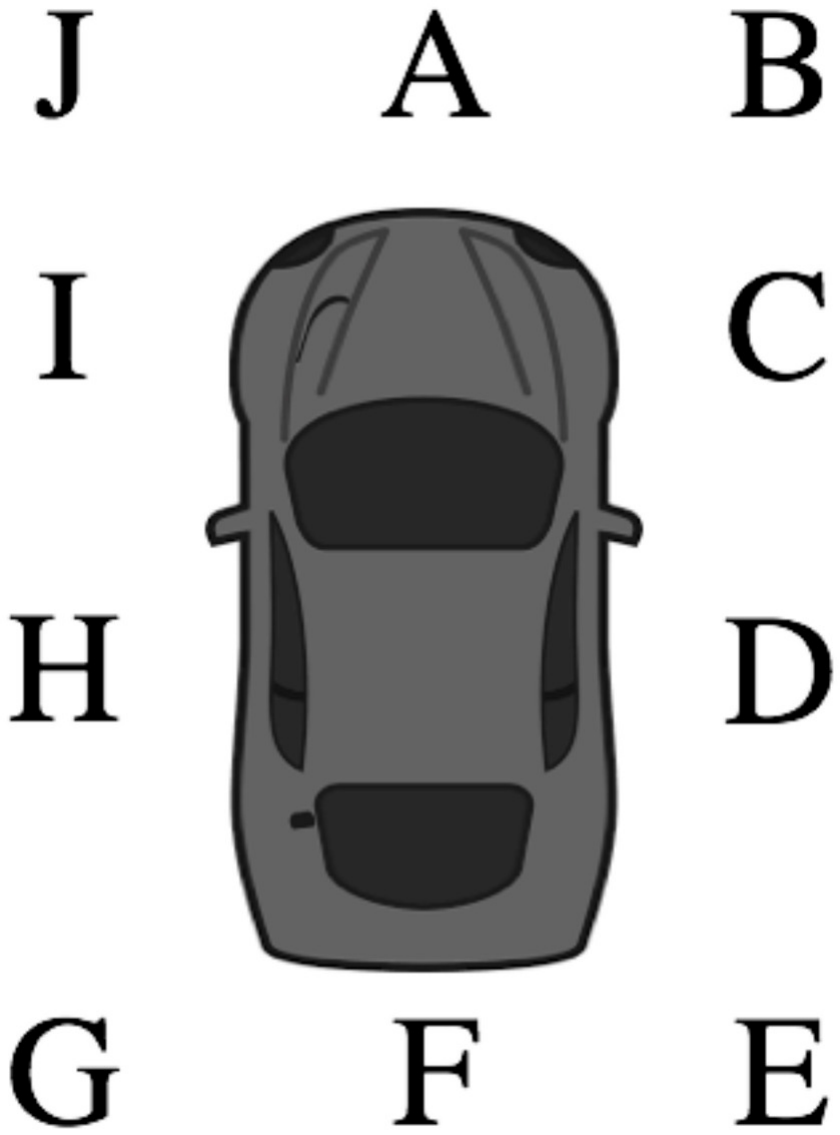
The subject vehicle is pictured. The position of impact is the location of the conflicting vehicle, person, animal, or object in relation to the subject vehicle. The position of impact was coded as one of ten (A–J) possible locations on the vehicle.

Previous research examining pseudoneglect in younger, middle, and older adults have identified changes in perceptual biases with age (Fukatsu et al., 1990; Varnava and Halligan, 2007; Schmitz and Peigneux, 2011; Benwell et al., 2014; Friedrich et al., 2016). Because rightward veering and collisions are thought to be associated with pseudoneglect (Turnbull and McGeorge, 1998; Nicholls et al., 2008), we hypothesized that age-related changes in navigation asymmetries would also be present. Additionally, given that rightward navigational asymmetries are repeatedly identified in laboratory experiments (Jang et al., 2009; Nicholls et al., 2010, 2016; Robertson et al., 2015), we hypothesized that the position of impact of crashes and near crashes would occur more frequently on the right side of the vehicle (see locations B, C, D, and E in Figure 1). We also hypothesized that the frequency of the rightward position of impact of crashes and near crashes would differ between younger and older adults, since laboratory experiments show a relationship between rightward deviations in navigation and perceptual biases, and since individuals who exhibit more frequent rightward collisions also demonstrate a larger leftward bias on the line bisection task (Turnbull and McGeorge, 1998; Nicholls et al., 2008).

## Method

### SHRP 2 NDS

To examine navigation asymmetries across the adult life span in a naturalistic setting, the frequency of the location of impact on the participants' vehicle during crashes and near crashes in a large sample of drivers from the SHRP 2 NDS was examined. The data retrieved from the SHRP 2 NDS were standardized variables that are outlined in the SHRP 2 Researcher Dictionary for Video Reduction Data (Virginia Tech Transportation Institute, 2015). Safety-critical events (e.g., crash, near crash) were classified based on kinematic and video analysis using automatic crash notification algorithms on the DAS, and controller area network algorithms on ingested data. These identified events were then reviewed on video by trained analysts, who categorized the events for severity and related characteristics, including precipitating events, evasive maneuvers, and position of impact. Details regarding the SHRP 2 NDS database and DAS instrumentation are outlined in Dingus et al. (2016).

The SHRP 2 NDS was sponsored by the Transportation Research Board (TRB) of the National Academy of Sciences. Initially, a website housing the data was accessed to determine the coded variables of interest for the study. Subsequently, to obtain user-access to the data, a Data Use License from the VTTI outlining variables of interest from the SHRP2 NDS was requested, following approval from the Behavioral Research Ethics Board of the University of Saskatchewan.

### SHRP 2 NDS variables examined

A number of variables related to the outcome of crashes and near crashes that occurred over the duration of the SHRP 2 NDS were examined. The SHRP2 Researcher Dictionary for Video Reduction Data (Virginia Tech Transportation Institute, 2015) identifies the outcome of events and incidents as a variable labeled *event severity*. Of the seven possible outcomes for event severity (crash, near crash, crash relevant, non-conflict, non-subject conflict, baseline, not applicable), crashes and near crashes were investigated. A crash was identified as any contact that the participant vehicle had with another object that was either moving or stationary. Road departures, where at least one tire left the roadway, were also considered crashes (Virginia Tech Transportation Institute, 2015). A near crash was identified as a circumstance that required a rapid evasive maneuver, either by the participant or any other vehicle, pedestrian, cyclist, or animal, to avoid a crash. In these circumstances a crash did not occur, and a non-premeditated, rapid, evasive maneuver (e.g., steering, braking, accelerating) was made to avoid the crash (Virginia Tech Transportation Institute, 2015). Over the course of SHRP 2 NDS, 1,465 crashes and 2,272 near crashes were identified, which were examined in the current study.

Of the 3,545 participants who participated in SHRP 2 NDS, 1,748 participants were involved in a crash and/or near crash. Demographic information for each participant was provided by the SHRP 2 NDS data set; however, the age of each participant in the data set was categorized into a 5-year age cohort. To ensure that a minimum of five crashes or near crashes occurred in each age group, 5-year age cohorts were combined to form 20-year age cohorts. Specifically, participants were separated into one of five age categories: 16–19 years, 20–39 years, 40–59 years, 60–79 years, and over 80 years of age. This categorization resulted in 332 (18.99%) participants between 16 and 19 years of age, 736 (42.11%) participants between 20 and 39 years of age, 270 (15.45%) participants between 40 and 59 years of age, 289 (16.53%) participants between 60 and 79 years of age, 97 (5.55%) participants above 80 years of age, and 24 (1.37%) participants who did not specify their age. Participants who did not specify his or her age were excluded. Of the 1,748 participants 841 (48.11%) were male, 896 (51.26%) were female, and 11 (0.63%) participants did not specify their sex. Participants who were 16–19 years of age had an average of 1.70 years of driving experience, participants 20–39 years of age had an average of 7.80 years of driving experience, participants between 40 and 59 years of age had an average of 33.58 years of driving experience, participants between 60 and 79 years of age had an average of 52.10 years of driving experience, and over 80 years of age had an average of 62.05 years of driving experience. On average, participants drove approximately 14,683.12 km per year. Table 1 compares the characteristics of the total SHRP 2 NDS sample and those who were involved in crashes and near crashes.

**Table 1:** Characteristics of the SHRP 2 NDS sample and participants who were involved in crashes and near crashes.

**Characteristic**	**Total sample (*n* = 3,545)**	**Crash/near crash sample (*n* = 1,748)**
Sex		
Male	1,668 (47.1%)	841 (48.1%)
Female	1,820 (51.3%)	896 (51.3%)
Missing	57 (1.6%)	11 (0.6%)
Age		
16–19	541 (15.3%)	332 (19.0%)
20–39	1,317 (37.2%)	736 (42.1%)
40–59	576 (16.2%)	270 (15.4%)
60–79	798 (22.5%)	289 (16.5%)
80 and above	225 (6.3%)	97 (5.5%)
Missing	88 (2.5%)	24 (1.4%)
Average annual mileage (km)	12,482.34	14,683.12
Previous years driving	27.17	22.34

The location of the other vehicle, pedestrian, animal, or object that was involved in the event, or that restricted the participant's ability to maneuver (i.e., in the participant's path of travel) at the precipitating event, was recorded in one of ten different locations (see Figure 1). The SHRP2 Researcher Dictionary for Video Reduction Data (Virginia Tech Transportation Institute, 2015) specifies that medians, barriers, and curbs were excluded and not considered to be objects in this category. If there was no motorist, non-motorist, animal, or object involved in the event, the location was categorized as *not applicable*, as there was no location to categorize (e.g., single-vehicle road departure, hitting a median, barrier, or curb). The location was coded as *unknown* if the position of the motorist/non-motorist could not be determined because of limitations in the video view, lighting, visual obstructions, or limited perspectives (Virginia Tech Transportation Institute, 2015). To ensure an adequate number of safety-critical events occurred in each location to complete statistical analyses, the location categories were reduced from ten to four by combining the locations on the right side of the vehicle (i.e., position B, C, D, E; see Figure 1) into a single right-side of the vehicle category and combining the locations on the left of the vehicle (i.e., position G, H, I, J; see Figure 1) into a single left-side of the vehicle category.

A unique advantage of a naturalistic driving study is the continuous monitoring of driving behavior, which provides detailed information preceding crashes. Variables from the SHRP 2 NDS data base that provide information preceding a crash included, the environmental state or the action by the participant, another vehicle, person, animal, or object that was critical to the participant being involved in a crash (i.e., precipitating event), the type of conflict the participant had with another object (i.e., incident type), and the participant's reaction or maneuver in response to the incident (i.e., evasive maneuver). Crashes of interest were therefore examined in further detail to determine the context in which the crash took place.

### Relative risk analyses

Relative risk examines a dichotomous variable and is calculated by comparing the probability of one event occurring to the probability of another event occurring (e.g., left vs. right crashes). The relative risk values calculated are greater than or equal to zero. A value of 1 indicates that the events are equally likely to occur, whereas a value greater or less than one indicates that an one of the outcomes is more or less likely to occur, respectively. The estimates of relative risk are accompanied by a lower and upper 95% confidence interval. Relative risk values are considered statistically significant if the confidence interval does not include 1.0. Further, when the relative risk upper and lower confidence intervals for a given age group are outside the upper and lower confidence intervals of any other age group, it can be taken with 95% confidence that there is a statistically significant difference in relative risk between the two age groups.

## Results

### Location of impact analyses

Tables 2, 3 outline the frequency and percentage (in parentheses) of the location of the conflicting vehicle, person, animal, or object in relation to the participants' vehicle for five age categories (16–19, 20–39, 40–59, 60–79, 80+) during crashes and near crashes, respectively. A high percentage of crashes did not involve a motorist, non-motorist, animal, or object, and did not have an applicable location to categorize (i.e., “not applicable” category). These were largely attributed to safety-critical events, including road departures that were classified as crashes but did not have an applicable location of impact on the vehicle (i.e., instances in which the participant's vehicle exited the roadway beyond the shoulder, beyond the end of the roadway, or onto the median). Given that there was no location of impact to analyze, these were excluded from further analysis, leaving 2,611 near crashes and 433 crashes to analyze.

**Table 2:** Frequency of position of impact in crashes.

**Age (years)**	**Right side of subject vehicle**	**Left side of subject vehicle**	**Front of subject vehicle**	**Rear of subject vehicle**	**Not applicable**	**Unknown**	**Total (N)**
16–19	30 (8.5%)	32 (9.0%)	56 (15.8%)	20 (5.6%)	216 (61.0%)	0 (0.0%)	354
20–39	46 (8.2%)	49 (8.7%)	70 (12.5%)	37 (6.6%)	359 (64.0%)	0 (0.0%)	561
40–59	6 (3.4%)	13 (7.3%)	15 (8.4%)	21 (11.7%)	123 (68.7%)	1 (0.6%)	179
60–79	14 (5.8%)	10 (4.1%)	25 (10.3%)	14 (5.8%)	177 (73.1%)	2 (0.8%)	242
80+	7 (6.1%)	10 (8.8%)	14 (12.3%)	7 (6.1%)	76 (66.7%)	0 (0.0%)	114

**Table 3:** Frequency of position of impact in near crashes.

**Age (years)**	**Right side of subject vehicle**	**Left side of subject vehicle**	**Front of subject vehicle**	**Rear of subject vehicle**	**Not applicable**	**Unknown**	**Total (N)**
16–19	95 (18.0%)	109 (20.6%)	282 (53.3%)	3 (0.6%)	40 (7.6%)	0 (0.0%)	529
20–39	340 (26.4%)	327 (25.4%)	574 (44.6%)	9 (0.7%)	37 (2.9%)	0 (0.0%)	1,287
40–59	111 (26.7%)	115 (27.6%)	182 (43.8%)	0 (0.0%)	8 (1.9%)	0 (0.0%)	416
60–79	130 (37.4%)	106 (30.5%)	105 (30.1%)	3 (0.9%)	4 (1.1%)	0 (0.0%)	348
80+	35 (31.8%)	37 (33.6%)	37 (33.6%)	1 (0.9%)	0 (0.0%)	0 (0.0%)	110

Table 4 shows the frequency of left and right crashes that participants in each of the five age categories were involved in, and the relative risk of crashing on the right and corresponding 95% confidence intervals. The confidence intervals for the relative risk for each age group was not outside the upper and lower confidence intervals of any other age group, suggesting the likelihood of a rightward crash was equal between the age groups. To specifically compare the age categories with the largest age difference, the relative risk of a younger (16–19 years) and older adult (over 80 years) crashing on the right was calculated. The relative risk of younger adults compared to older adults crashing on the right was 0.89, 95% CI [0.40–1.99], indicating that younger and older adults had an equal risk of crashing on the right. Further, examination of the relative risk for each age group revealed that only 16–19-year-old participants had a statistically significant difference between the frequency of left and right crashes. Participants in the youngest age group were 0.52 times as likely to crash on the right compared to the left, 95% CI [0.29–0.94]. A statistically significant difference between the frequency of left and right crashes was not found among the other four age groups (see Table 4).

**Table 4:** Frequency of position of impact during crashes without road departure incidents, and relative risk of crashes on the right side of participants' vehicles with corresponding 95% confidence intervals.

**Age (years)**	**Right side of subject vehicle**	**Left side of subject vehicle**	**No. of crashes**	**Relative Risk of rightward crash**	**Lower confidence interval**	**Upper confidence interval**
16–19	14	27	112	0.52	0.29	0.94
20–39	33	41	173	0.80	0.54	1.21
40–59	6	11	59	0.55	0.22	1.38
60–79	10	9	39	1.11	0.51	2.43
80+	5	8	50	0.63	0.22	1.78

Similarly, Table 5 shows the frequency of left and right near crashes that participants in each of the five age categories were involved in, and the relative risk of crashing on the right and corresponding 95% confidence intervals. Again, the relative risk upper and lower confidence intervals for each age group were not outside the upper and lower confidence intervals of any other age group, suggesting that each age group had an equal risk of crashing on the right side of the vehicle. Additionally, the 95% confidence intervals for the relative risk calculation in each age group included 1.0 and did not reveal a statistically significant difference between the frequency of left and right near crashes (see Table 5).

**Table 5:** Frequency of position of impact during near crashes without road departure incidents, and relative risk of near crashes on the right side of participants' vehicles with corresponding 95% confidence intervals.

**Age (years)**	**Right side of subject vehicle**	**Left side of subject vehicle**	**No. of near crashes**	**Relative risk of rightward near crash**	**Lower confidence interval**	**Upper confidence interval**
16–19	94	106	497	0.89	0.69	1.14
20–39	337	326	1,255	1.03	0.91	1.18
40–59	111	114	406	0.97	0.78	1.22
60–79	129	105	343	1.23	1.00	1.52
80+	35	37	110	0.95	0.65	1.38

Overall, examining the frequency of crashes on the right (*n* = 68) and left (*n* = 96) of participants' vehicles revealed a statistically significant difference. However, unlike results from experiments examining navigation asymmetries in laboratory environments, leftward crashes were significantly more frequent than rightward crashes. Crashes were 1.41 times as likely to occur on the left compared to the right side of participants' vehicles, 95% CI [1.07–1.87].

### Characteristics of crashes

Of the 96 crashes that occurred on the left side of the participants' vehicle, 89 (92.7%) occurred in position J (see Figure 1). Crashes on the left were most often preceded by the participant turning left at an intersection (16.7%), an animal on the roadway (15.6%), and another vehicle entering the intersection straight across the participant's lane of travel (10.4%). These precipitating events are consistent with the most common types of conflicts (i.e., incident types). The types of conflicts most common when crashes were on the left of the participant's vehicle were contact with a living animal (20.8%), the participant or other vehicle crossed in front of the other vehicle when turning left or right (16.7%), and interactions that were not coded in one of the other 18 incident type categories (15.6%). In an attempt to avoid the crash, the most common reactions and maneuvers (i.e., evasive maneuver) were braking that resulted in skidding (33.3%), braking and steering right (26.0%), and no reaction or change in driving behavior (26.0%).

Sixty-eight crashes occurred on the right side of the participants' vehicle. Crashes on the right were most often preceded by an animal approaching the roadway (11.8%), an animal on the roadway (8.8%), another vehicle entering the intersection straight across the participant's lane of travel (5.9%), and participant backing their vehicle (5.9%). The types of conflicts (i.e., incident types) that most common occurred when crashes were on the right of the participant's vehicle were contact with a living animal (20.6%), interactions that were not coded in one of the other 18 incident type categories (20.6%), and turned into path of another vehicle (11.8%). To avoid the crashes, the most common reactions and maneuvers (i.e., evasive maneuver) were braking with no brake lockup (26.5%), no reaction or change in driving behavior (23.5%), and braking and steering left (20.6%).

**Figure 2:**
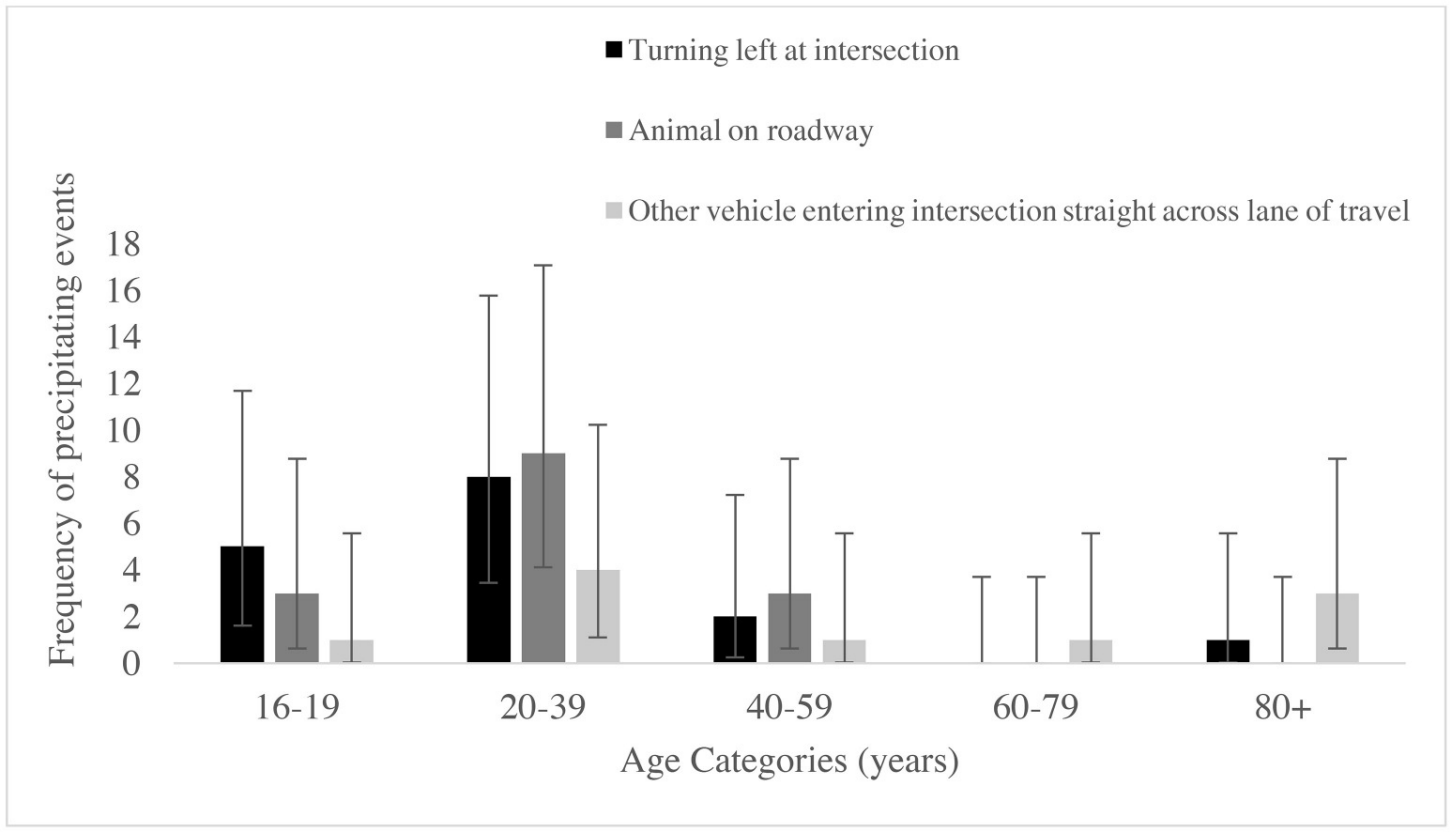
The frequencies and 95% Poisson confidence intervals of the most common precipitating events preceding crashes on the left of participants' vehicles in the five age categories.

**Figure 3:**
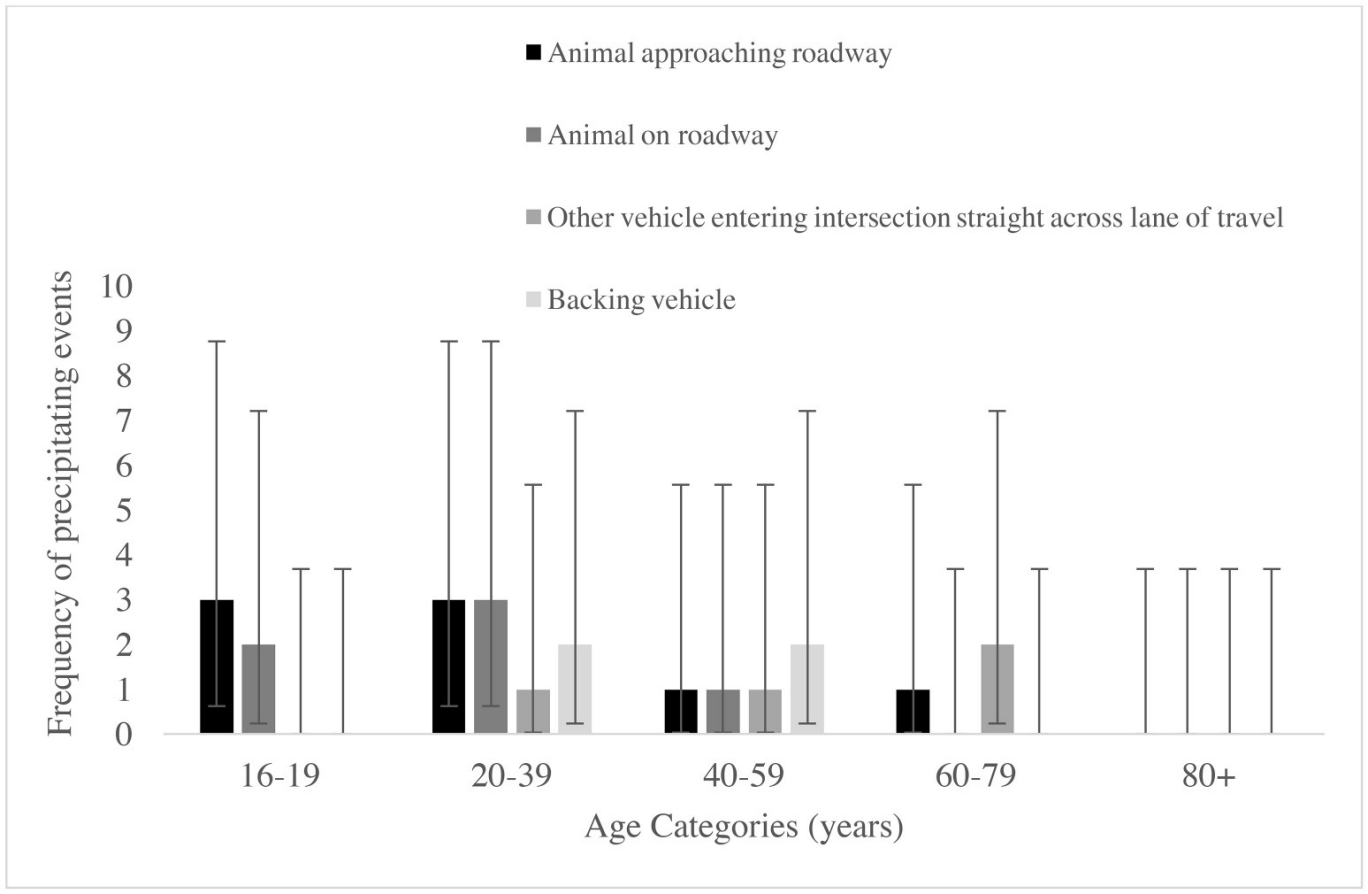
The frequencies and 95% Poisson confidence intervals of the most common precipitating events preceding crashes on the right of participants' vehicles in the five age categories.

Although crashes on both the left and right of participants' vehicles were often commonly preceded by a conflict with an animal, there was a difference in common scenarios preceding crashes that potentially involved human error. Crashes that occurred on the left were commonly preceded by turning left at an intersection, whereas crashes on the right occurred more often prior the participant backing their vehicle or another vehicle entering the intersection and traveling across the participant's travel lane. However, due to the low frequency of precipitating events we were unable to statistically compare the frequency of the common precipitating events, or across age groups. Rather, the frequency of precipitating events on the left and right of participants' vehicles in each age group are displayed graphically (see Figures 2, 3).

## Discussion

The aim of the study was to examine if age-related changes in pseudoneglect are also present in navigation asymmetries, and whether navigation asymmetries found in laboratory environments are present while driving in a naturalistic setting. Prior research examining asymmetry in navigation has primarily examined younger adults through retrospective reports or through experiments in controlled laboratory settings. Findings from these experiments has consistently identified veering asymmetries that result in small (10–36 mm), but consistent deviations to the right (Jang et al., 2009; Nicholls et al., 2010, 2016; Robertson et al., 2015). These small, systematic deviations in a controlled environment have been proposed to result in collisions in naturalistic settings (Nicholls et al., 2016) where navigation is more complex (e.g., parking a car in parkades or garages, and driving over narrow bridges). This study is the first (to our knowledge) to extend laboratory research, and examine the association between age-related changes in pseudoneglect and navigation asymmetry using crash analysis in a naturalistic setting. Data available from the SHRP 2 NDS resulted in examining the location of position of impact during crashes and near crashes, which is distinct from measures of veering that can identify subtle asymmetries when navigating. However, when examining a large sample and 35 million vehicle miles, it was hypothesized that subtle rightward biases in veering would result in a greater number of rightward compared to leftward crashes and near crashes.

Despite previous observational studies documenting rightward veering when walking (Nicholls et al., 2007, 2008), navigating an electric vehicle through a doorway (Nicholls et al., 2010, 2016; Robertson et al., 2015), or while driving a car in a driving simulator (Jang et al., 2009), the location of impact on the participants' vehicle during crashes and near crashes did not occur more frequently on the right side. In contrast, during crashes the other vehicle, non-motorist, animal, or object was more likely to be located on the left side of the vehicle. Of the age groups examined, participants 16–19 years of age had a greater risk for leftward crashes. Further, in contrast to previous laboratory experiments that have identified age-related changes in pseudoneglect, it is unclear whether age was related to position of impact due to the null association between age category and position of impact. Before conclusions are drawn regarding whether asymmetries in navigation are present in naturalistic settings, and whether age is related to navigation asymmetry, additional research is needed. The following explanations could account for our contradictory and null findings, including the measure of navigation asymmetry used, complexity of left turns, and allocation of attention.

One of the most likely reasons for the weak findings of asymmetry in the position of impact during crashes and the lack of asymmetry in near crashes is the sensitivity of the outcome measure. Veering to the right when walking and navigating electric wheelchairs, scooters, and miniature vehicles is a subtle, but systematic, asymmetry that may not be detected when examining the location of the impact on the participants' vehicle during crashes and near crashes. Researchers extending laboratory findings of asymmetries in navigation to a naturalistic setting may benefit from using sensitive measures of asymmetry while driving, such as lane position data. Tracking where participants drive within their lane could provide data akin to veering in laboratory environments. Radar located on the vehicles collected lane positioning data, however, at the time of the analysis, such data was not available from the SHRP2 NDS database.

The measure of navigation asymmetry may have also impacted the crash and near crash symmetry demonstrated by each age group. From our analysis and the null association between age category and position of impact, it appears that age is not related to position of impact, as the symmetry of crashes and near crashes on the left and right side of participants' vehicles was consistent across the five age groups examined. This empirical observation is at odds with the notion that younger adults demonstrate rightward veering when navigating (Nicholls et al., 2010, 2016; Robertson et al., 2015) and that older adults typically fail to demonstrate the presence of pseudoneglect compared to younger adults (Fukatsu et al., 1990; Fujii et al., 1995; Failla et al., 2003; Barrett and Craver-Lemley, 2008; Goedert et al., 2010; Chen et al., 2011; Schmitz and Peigneux, 2011; Benwell et al., 2014). It is hypothesized that utilizing a measure with enhanced sensitivity to navigation asymmetry would assist in examining the subtle attenuation or intensification of pseudoneglect as participants age.

Certainly, the availability of more precise data about navigational asymmetries will not change the fact of the crashes themselves. What it will help with is understanding competing explanations for the crashes, including attentional bias effects, driving experience effects, effects of the driving environment (e.g., traffic directionality, driving environment), and interactions among these effects. For example, a possible explanation for the unexpected finding of a greater frequency of crashes on the left of participants' vehicles, is the complexity of left turns. Examining circumstances that precipitate crashes gives an indication of actions that made the crash possible. The SHRP2 Researcher Dictionary for Video Reduction Data (Virginia Tech Transportation Institute, 2015) identified 76 possible precipitating events. Of the actions that compare left and right turns (e.g., turning left or right at an intersection, departing a lane to the left or right), participants turning left from its roadway to another roadway resulted in the highest percentage of crashes on the left of the participants' vehicle (16.7%), whereas turning right from its roadway preceded 3.1% of crashes. As expected, the evasive maneuver (i.e., drivers' reaction) in response to the event or incident was most often to brake and steer to the right of initial travel direction (26%) in an attempt to avoid a crash on the left side of the vehicle. Braking and steering to the left of the initial travel direction occurred in 3.1% of crashes on the left of the participants' vehicle.

In countries, such as the United States of America, which have right-sided traffic directionality (i.e., citizens drive on the right side of the road), left turns require greater attention to and observation of the left hemifield, compared to left-sided traffic directionality (Foerch and Steinmetz, 2009). When turning left, attention is shifted rightward, and motorists may have difficulty attending to other motorists and non-motorists in oncoming traffic who are located in the left visual field. The bisection model of navigation asymmetries proposes that rightward veering and collisions results from participants moving toward the perceived center (i.e., right of true center) without updating their trajectory when moving toward a target or aperture (Nicholls et al., 2010). The theory has been supported by eye tracking data gathered during navigation tasks that have identified mean eye position to the right when moving a wheelchair through an aperture (Robertson et al., 2015), and positive associations between perceived midpoint of an aperture and where the vehicle passed through the aperture (Nicholls et al., 2016). Thus, the high proportion of crashes during left turns and the higher frequency of leftward crashes in the SHRP2 NDS may be explained by the rightward attentional bias, as participants' attention may have been shifted rightward during left turns.

An alternative explanation for the overall leftward bias during crashes is allocation of attention in the upper or lower visual field due to the visual environment (Hatin et al., 2012). Location of the stimuli in the upper or lower visual field has been found to modulate the directional bias in collision behavior (Thomas et al., 2009). Further, when navigating, research has proposed that participants are biased to move toward locations where their attention is directed rather than moving away from attended areas (Nicholls et al., 2010; Hatin et al., 2012), as drivers have been found to have a tendency to steer in the direction they are looking, even when it is dangerous to do so (Wilkie et al., 2010). Together, shifts in vertical allocation of attention and moving toward attended areas may result in collisions on the side that is attended (Hatin et al., 2012).

Laboratory experiments that involve navigating an electric vehicle through a doorway (Nicholls et al., 2010, 2016; Robertson et al., 2015) direct participants' attention downward. Downward shifts in attention to the lower visual field have also been associated with shifts in attention to the right visual field over the left visual field (Nicholls et al., 2004), which has been suggested to result in rightward collisions (Hatin et al., 2012). In contrast, attention to the upper visual field has been associated with biases to the left visual field (Nicholls et al., 2004). Biases in attention to the upper-left visual field have been supported in a number of studies. For example, targets are identified significantly faster when they appear to be lit from the upper-left (Sun and Perona, 1998; McManus et al., 2004) and when they are located in the upper-left quadrant (Smith et al., 2014) compared to other lighting directions and locations. Leftward biases are also strongest during the line bisection task when the lines are presented in the upper visual field (McCourt and Jewell, 1999; McCourt and Garlinghouse, 2000). Consequently, variations in the vertical visual field in which the task is carried out may account for differences in the direction of collisions between previous research in laboratory environments and the current findings.

Unlike previous research that has examined navigation asymmetries where participants may have been biased to direct their attention downwards, participants in the SHRP2 NDS may direct their attention to the upper visual field when driving in a naturalistic setting. For example, while driving a vehicle, the roadway is likely in the participant's upper visual field, whereas the dashboard of the vehicle is in his or her lower visual field. As a result, participants' attention may be biased to the upper left visual field, as found in previous research (Nicholls et al., 2004), resulting in a shift in attention to the left, leading to a leftward crash bias. In contrast to hypothesizing that biases in collisions are associated with pseudoneglect or perceptual asymmetries, biases in collisions to the left or right may also result from situational variables that influence the allocation of attention to the upper or lower visual field.

### Limitations

In an attempt to enhance the external validity of laboratory research examining navigation asymmetry, in the present study we examined the position of impact following crashes and near crashes in a naturalistic setting. The naturalistic driving data used provided the prevalence of crashes and near crashes at different positions on participants' vehicles and pre-collision information, which allowed examination of the association between the lateralized behavior and pseudoneglect in a real-world environment. However, utilizing data from a naturalistic driving study involves methodological limitations, particularly with regards to confounds and noise in the data, as we were unable to control the variables examined (Carsten et al., 2013). For example, the frequency of crashes and near-crashes on the left or right side of the participants' vehicle may have been influenced by additional variables such as, the overall frequency of left and right turns—a variable that we were unable to examine. Naturalistic driving studies also focus on the human element in event causation, which limited our ability to examine traffic-system-based problems and the role of other drivers, pedestrians, or animals in the frequency of crashes. As a result, we cannot isolate a cause and effect relationship between the variables, but are able to discuss observed associations. Further, because data from naturalistic driving studies are used for a broad range of research questions, we were limited to the non-parametric retrospective nature of the data (i.e., a safety critical event are identified first and contributory factors are examined second) and the data collected. For instance, age was provided as a categorical variable, we were unable to examine the number of attempted turns, and, as mentioned above, data regarding participants' lane position was unavailable. Nonetheless, traffic safety is complex issue and examining real-world behavior contributes to the literature examining navigation asymmetries in controlled environments. The use of naturalistic driving data also provided objective pre-collision characteristics (e.g., common precipitating events) that can be used to generate new hypotheses and subsequently tested under controlled conditions such as, test-tracks or driving simulators (Carsten et al., 2013).

## Conclusion

The current study and findings from the SHRP2 NDS add to the growing body of research on navigation asymmetry. The present investigation documents an overall leftward collision bias and a failure to find a difference in a collision bias between age groups. These findings are in contrast to rightward collisions predicted by the pseudoneglect hypothesis and previous results demonstrated in laboratory experiments. Extending laboratory research findings to naturalistic settings enhances the external validity of results, and informs future research of the complexities and limitations associated with naturalistic observation research. Utilizing measures that are not sensitive enough to examine asymmetries in navigation, the complexity of driving in natural settings, and allocation of attention to the upper visual field may account for the disparities among rightward collisions reported in the literature and the current results. Researchers who conduct future research in naturalistic settings would likely find utility in examining lane positioning data that has the ability to examine subtle changes in veering, as well as crash and near crash data to enhance the understanding and practical impact of asymmetries in navigation.

## Ethics statement

The SHRP2 NDS was sponsored by the Transportation Research Board (TRB) of the National Academy of Sciences. All subjects gave written informed consent. The Behavioral Research Ethics Board of the University of Saskatchewan approved the requested variables from the SHRP2 NDS.

## Author contributions

The grant that supported the manuscript was awarded to LE. TF obtained the data set and analyzed the data. TF, LE, and PH wrote the manuscript.

### Conflict of interest statement

The authors declare that the research was conducted in the absence of any commercial or financial relationships that could be construed as a potential conflict of interest.
